# Investigation of Real-Time Diagnostic Ultrasound as a Means of Biofeedback Training in Transversus Abdominus Re-Education of Patients with Non-Specific Low Back Pain: A Prospective Randomized Controlled Pilot Study

**DOI:** 10.3390/healthcare11101396

**Published:** 2023-05-11

**Authors:** Nikolaos Taxiarchopoulos, Elena Drakonaki, Maria Gianniotis, Charalampos Matzaroglou, Elias Tsepis, Evdokia Billis

**Affiliations:** 1 Department of Physiotherapy, University of Patras, 26504 Rion, Greece; margiannioti@yahoo.gr (M.G.); matzaroglou@upatras.gr (C.M.); billis@upatras.gr (E.B.); 2 Medical School, University of Crete, 71003 Heraklion, Greece

**Keywords:** low back pain, motor control exercises, real-time ultrasound imaging

## Abstract

**Background:** It is believed that ultrasound-guided imaging of activation/contraction of the deep abdominal muscles (such as transervsus abdominis) is useful for assisting deep muscle re-education, which is often dysfunctional in non-specific low back pain (NSLBP). Thus, this pilot study aimed to evaluate the use of real-time ultrasound (US) as a feedback device for transverse abdominis (TrA) activation/contraction during an exercise program in chronic NSLBP patients. **Methods:** Twenty-three chronic NSLBP patients were recruited and randomly assigned to a US-guided (*n* = 12, 8 women, 47.6 ± 2.55 years) or control group (*n* = 11, 9 women, 46.9 ± 4.29 years). The same motor control-based exercise program was applied to both groups. All patients received physiotherapy twice per week for seven weeks. Outcome measures, tested at baseline and post-intervention, included Numeric Pain Rating Scale, TrA activation level (measured through a pressure biofeedback unit-based developed protocol), seven established motor control tests, Roland-Morris Disability Questionnaire and Hospital Anxiety and Depression Scale. **Results:** For each group, all outcome variables yielded statistical differences post-intervention (*p* < 0.05), indicating significant improvements. However, there were no significant group x time interactions for any of the outcomes (*p* > 0.05), thus, indicating no superiority of the US-guided group over the control. **Conclusions:** The addition of US as a visual feedback device for TrA re-education during a motor control exercise program was not proven superior to traditional physiotherapy.

## 1. Introduction

Non-specific low back pain (NSLBP) is one of the most common conditions affecting general and specific population samples worldwide [1,2]. Low back muscle dysfunction has been the subject of numerous studies, associated with chronic NSLBP. The muscles believed to contribute significantly to trunk ‘stability’ are the deeper trunk muscles, such as transversus abdominis (TrA) and multifidus, and some researchers have shown that altered activation patterns of deep trunk muscles when present, are related to lack of spinal stability [3,4,5]. 

In individuals with NSLBP, TrA contraction has been reported to be significantly delayed in activation during a physical task or movement, indicating a potential for reduced spinal stability as well as underlying motor control problems in the lumbopelvic region [6]. Indeed, it has also been reported by Panjabi [7] that both, multifidus and TrA have a greater role than other deep muscles in lumbar stability, while at the same time demonstrating a reduced cross-sectional area in patients with chronic NSLBP [8,9]. In individuals with NSLBP, the local musculature exhibits impaired motor control resulting in altered normal muscle activation patterns [3,5,10,11,12,13]. Specifically, Lamoth et al. [14] presented altered motor control patterns as a cause of increased chronicity and recurrence of low back pain symptoms. Motor control exercises (MCE) are suggested to correct such deficits and retrain optimal motor patterns as well as spinal motion control and are currently used by physiotherapists worldwide for the management of NSLBP [15].

Individual activation of the deep abdominal muscles appears to be particularly difficult for individuals with NSLBP. Studies in NSLBP subjects have reported overuse of the rectus abdominis [16], as well as altered recruitment patterns of TrA [3]. TrA is deeply located and cannot be palpated in isolation. To address the difficulties of teaching and learning the activation/contraction of deeper muscles, several physiotherapy researchers and clinicians advocate supplementing traditional feedback methods (i.e., verbal and/or palpatory cues) with the use of real-time ultrasound imaging to provide increased visual feedback for optimal effect [17]. Ultrasound (US) images of the abdominal wall can provide accurate visual feedback and instant knowledge of performance to the patient by projecting the contraction (muscle movement) of the subject’s deeper abdominal muscles on the ultrasound screen in real time [18]. 

Thus, given the above, the purpose of this study was to explore whether combining standard clinical education for TrA activation with real-time US-guided feedback is more effective than traditional physiotherapy, while performing the same motor control exercise-based exercise program in people with NSLBP.

## 2. Materials and Methods

### 2.1. Setting and Participants

A single-blind randomized controlled pilot trial with two intervention groups was conducted during a four-month period. People with chronic NSLBP with/or without related referred lower extremities symptoms were sought from the broader Achaia region via an open invitation from the Physiotherapy department, which was made public through social media and local press. Volunteers were selected if they were adults aged 18–60 years old and suffered from NSLBP lasting longer than 12 weeks, producing moderate or severe disability [19,20,21]. Excluded from the study were people with previous spinal surgery, people suffering from systemic diseases and pregnant women [22,23,24].

The research protocol was approved by the Ethics Committee of the University of Patras (protocol number 12614). Prior to the commencement of the study, all participants signed an informed consent form.

### 2.2. Interventions

The intervention program lasted 7 weeks with a frequency of 2 individual sessions per week, each session lasting approximately 30–40 min. The motor control exercise rehabilitation program was developed through the literature and consisted of three phases; warm-up, main program and recovery phase [8,25,26,27]. Warm-up included 6 exercises (3 sets × 10 repetitions each), aiming to activate the structures to be used, to enhance performance [28]. The main program consisted of a total of 11 exercises of progressive difficulty. In each treatment session, 6 exercises (3 sets × 10 repetitions each) were performed [29,30]. Regarding progression, in the first two weeks exercises were performed from a lumbar non-loading position (crook lying position); in the next two weeks and as long as the patient could correctly perform the most difficult exercises (10 repetitions with a 10 s hold each), a progression was applied from other positions, such as quadruped, then sitting, and finally standing. Recovery included 4 static self-stretching exercises, 2 to 4 repetitions each, lasting for 60 s [31,32]. A summary of the exercise program is provided in Appendix A. Both (control and intervention) groups received the same therapeutic exercise program. Participants were not allowed to receive other treatments for NSLBP during the intervention period. 

#### 2.2.1. Control Group 

The control group received traditional tactile feedback from the therapist while performing the motor control exercises for TrA activation. Traditionally, assessment of TrA contraction involves palpation of the muscles [5,33,34]. The ability to assess TrA through muscle palpation has moderate reliability [33] and is largely dependent on the examiner’s skill, as TrA cannot be directly palpated (feedback sensation being limited from internal oblique muscle) [18,33]. To control the activation of the abdominals the therapist placed his hands on the inside of the anterior iliac crests (tactile feedback) and instructed the examinee to pull the abdominal wall inward without moving the spine or pelvis (verbal feedback).

#### 2.2.2. US-Guided Intervention Group 

**Figure 1:**
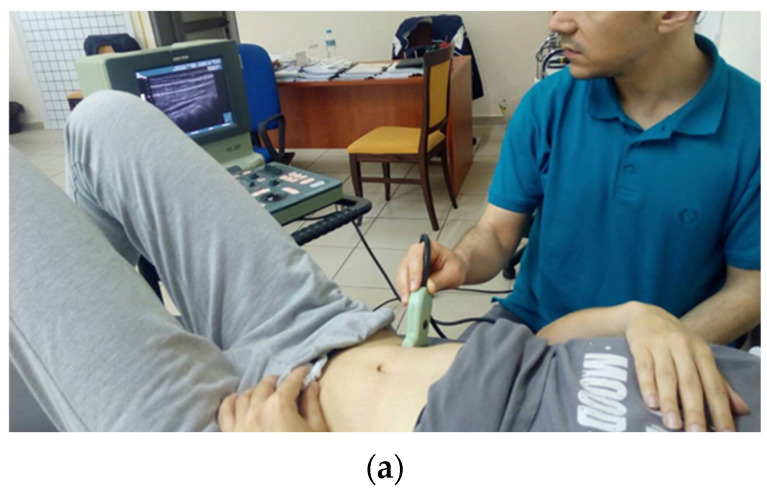

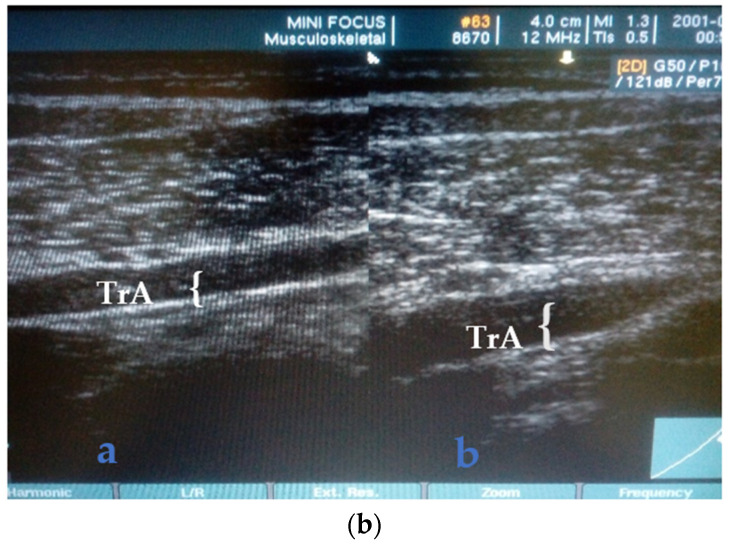
**Transversus abdominis activation training with visual feedback using US**. Positioning of the patient and US probe (**a**). US imaging of the outer abdominal wall (**b**) Imaging of the abdominal wall at rest (left) and during an isolated TrA contraction (right), showing an increase in the width of the TrA.

This group received visual feedback using real-time US for TrA activation, while performing the exercises (Figure 1). Τhe ultrasound equipment used was B-K Μedical Mini Focus 1402 equipped with high frequency linear probe (8670, 5–12 MHz) using standard musculoskeletal settings and the software: V 1.01.01.137. Ultrasound gel (AQUASONIC^®^ 100, Parker Inc., Orange, NJ, USA) was used as a coupling agent. TrA imaging was initially performed with participants in crook lying, with the US head positioned along the lateral abdominal wall with reference points at the lower point of the rib cage (last rib) and the anterior superior iliac spine, on the right side of the person, midway between these two points [34,35]. The US head was moved until we had the best possible visualization of the lateral abdominal muscles (external oblique, internal oblique and TrA) [36]. During the execution of the exercises, the participants could watch the ultrasound screen along with the therapist’s guidance, thus receiving information (US-visual feedback) about their TrA activation (Figure 1). 

### 2.3. Outcome Measures

#### 2.3.1. Patient-Reported Measures 

Three popular for NSLBP self-reported questionnaires were used; Numerical Pain Rating Scale (NPRS) for measuring pain intensity [37], Roland-Morris Disability Questionnaire (RMDQ), a 24-tem self-reported measure of low back pain-related disability [38] and Hospital Anxiety and Depression Scale (HADS), for identifying anxiety and depression [39], since anxiety and/or depression often co-exist in chronic NSLBP populations and subsequently, may hinder recovery.

#### 2.3.2. Clinician-Reported Measures

##### Motor Control Tests

**Figure 2:**
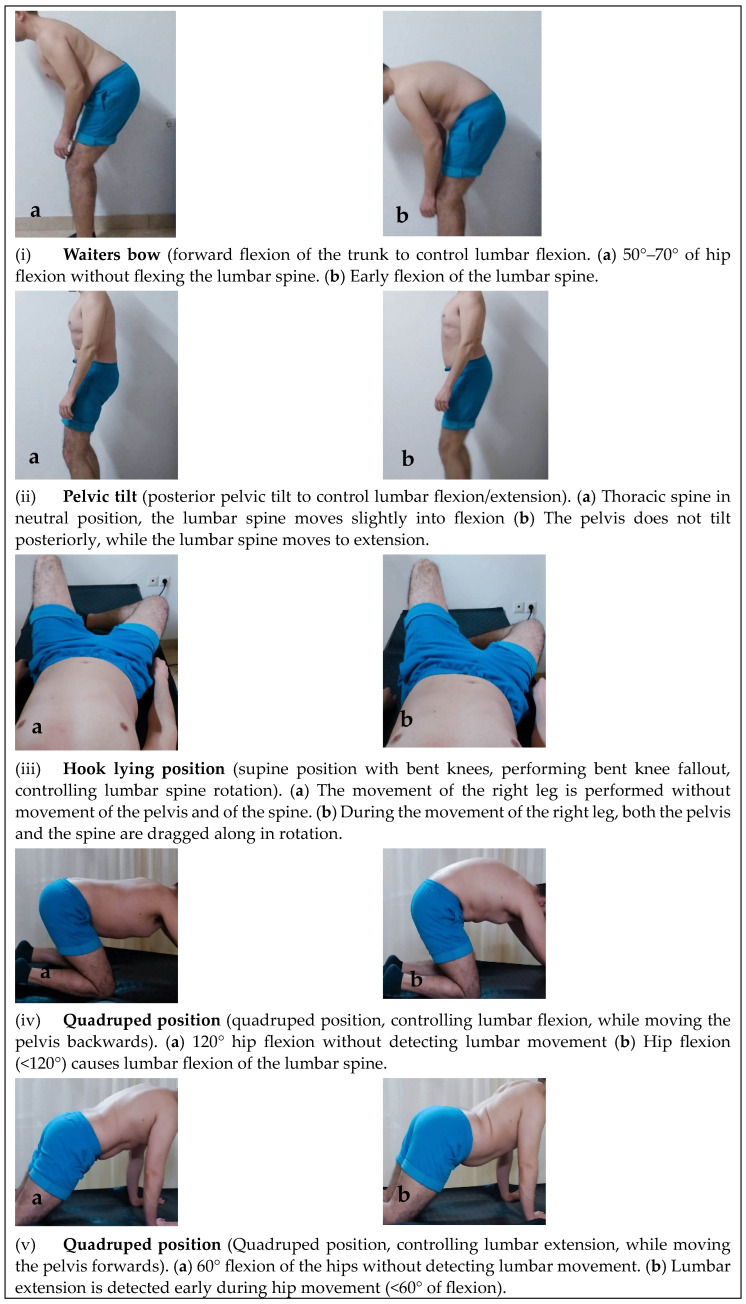

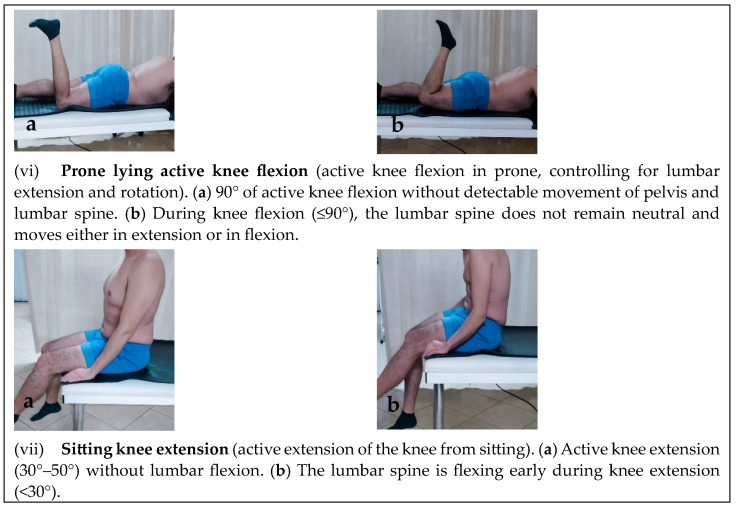
Motor control tests; where, the images on the left (**a**) show the correct way of performing test while the images on the right (**b**) show the incorrect way.

Seven reliable motor control tests (Figure 2) previously utilized with chronic NSLBP populations were used [29,40]. These were: (i) waiters bow, (ii) pelvic tilt, (iii) hook lying position, (iv) quadruped position (flexion-control), (v) quadruped position (extension-control), (vi) prone lying active knee flexion and (vii) sitting knee extension. An inter-tester reliability procedure for these tests was conducted prior to data collection. Measurements were performed by two trained physiotherapists on 10 subjects. Each subject was measured first by one therapist and then by the other, whistle the order of the therapists was random. A second measurement was performed one week after the first one. Physiotherapists visually evaluated the movement quality of these tests, marked as correct or incorrect, as indicated in relevant literature [29,40]. Inter-tester reliability was estimated on all assessment attempts of the therapists on each subject. 

##### TrA Activation and Standardization of Exercise Progression Level

We developed a progressive assessment procedure for TrA activation and exercise progression sequence using the stabilizer pressure biofeedback unit (Chattanooga Group model, US). This device is extensively popular and reliable for assessing, monitoring and feedback on deep abdominal musculature, such as TrA by measuring a change in pressure during abdominal muscle contraction [41,42,43].

By placing the biofeedback device on the subject’s back whistle supine and utilizing specific verbal instructions along with tactile feedback for activating TrA, the pressure being produced on the biofeedback during muscle contraction, ascertains whether the patient is able to activate and/or sustain contraction of the muscles [3,44,45,46]. Progressively, the difficulty is increased by adding more repetitions or longer contraction holds or limb movements. 

For the study’s purposes, a pre-pilot was conducted in 10 people (5 with and 5 without chronic NSLBP), to assist in developing and standardizing the exercise progression levels [47,48]. For this purpose, an exercise selection in the crook lying position (with 80–90° of hip flexion and 45° of knee extension) was chosen. All patients had visual feedback by monitoring the manometer on the pressure biofeedback device. Initially, the feedback device was inflated to a pressure of 40 mmHg. By utilizing specific instructions [44,45,46], each TrA contraction was requested to be activated and last 10 s, while the acceptable pressure deviation during the execution of each contraction was required to be within 2 to 4 units mmHg change (between rest and contraction). The activation levels for allowing exercise progressions to take place were determined as follows:

Level 1: Single contraction of the TrA, lasting 10 s

Level 2: Three TrA abdominal contractions, lasting 10 s

Level 3: Ten abdominal TrA contractions, lasting 10 s

Level 4: Holding the TrA contraction while performing 5 repetitions of side bending (and return) of each leg (bend knee fallout and return)

Level 5: Holding the TrA contraction while performing 5 bend leg raises on each leg

Level 6: Holding the TrA contraction while performing 5 repetitions of bending and extending each leg (leg slides).

Each participant was instructed to relax his/her whole body, especially the abdominals, before each contraction. He/she was asked to perform a submaximal contraction of the TrA for one repetition lasting 10 s and so on (in accordance with the biofeedback unit’s requested measurement changes), whistle a 1-min break between each testing level was given. Each participant’s assessment level was defined as that level at which he/she could complete the required repetitions successfully and with relative ease. Inter-tester reliability was also tested between the two therapists undertaking the assessment on two subsequent measurements of this procedure (for each subject).

### 2.4. Randomization

Participants were randomly assigned to one of two treatment groups; those who received ultrasound-guided feedback during their exercise intervention (US-guided group) and those who received the traditional exercise intervention, without ultrasound guidance feedback (control group). Participants were tested at baseline and post-intervention. Eligibility was assessed by a research physiotherapy assistant, who was blind to the scope of the study. To divide individuals into one of the groups, the method of block randomization in blocks of four was chosen (to ensure equal number allocation).

### 2.5. Data Analysis

Τhe intraclass correlation coefficient (ICC_1,2_) was used in order to measure inter-tester reliability of the TrA activation level and the motor control tests. Analysis of variance utilizing a two-way mixed analysis of variance (ANOVA) model for dependent measures of two factors (treatment group and time point of measurement) of which only one is repeated (time point of measurement) was performed to determine whether there were between- and within-group differences before and after treatment. Independent samples’ t-tests and paired samples’ t-tests were also used for differences between and within groups, respectively. An χ^2^ test was also conducted for differences in the motor control tests and the TrA activation level procedure across the groups. SPSS 25.0 statistical package was used to analyze the data. The level of significance was set at *p* < 0.05.

## 3. Results

**Figure 3:**
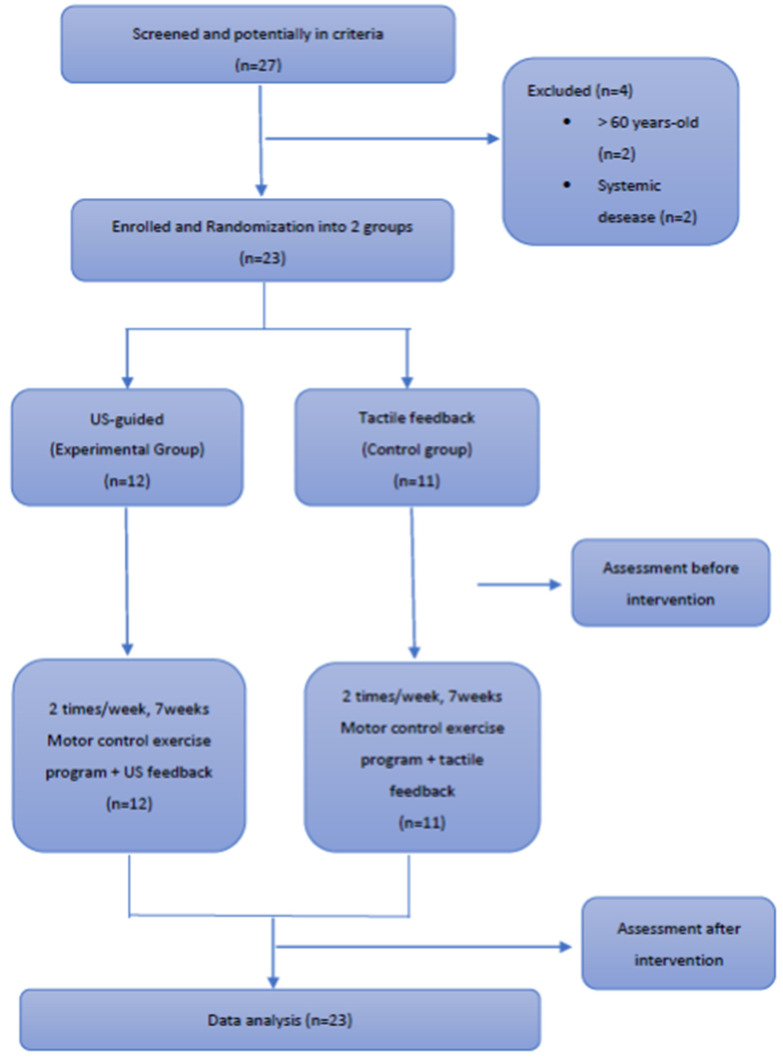
Consort Flow diagram summarizing participant flow in the study.

Of 27 subjects with NSLBP who were evaluated, 23 (17 women, 6 men) met the inclusion criteria for the study; four individuals were deemed ineligible (2 were older than 60 years old and 2 had systemic diseases). The 23 participants were randomly assigned into two groups, the intervention (US-guided) group (*n* = 12, 8 women, 4 men, aged 47.6 ± 2.55 years) and the control group (*n* = 11, 9 women, 2 men, aged 46.9 ± 4.29 years). No statistically significant differences were found on baseline (*p* > 0.05) across all variables. No dropouts were reported as all 23 managed to attend and complete the intervention. Figure 3 summarizes the Consort flow diagram for the study. Participants’ demographic and clinical characteristics are listed in Table 1. The total sample (Table 2) and across groups results (Table 3 and Table 4) are summarized below. 

Results from frequency analysis regarding the whole sample, indicated that there are statistically significant differences in all outcomes following the motor control intervention (except for HADS anxiety, in which there were no statistically significant differences, Table 2).

However, two-way mixed ANOVA results did not report statistically significant differences between the two groups (US-guided versus control) in any of the outcomes of pain, disability, anxiety or depression measured following the intervention (Table 3). Additionally, chi square analysis between the groups post-intervention also yielded non-significant differences across the groups on the TrA biofeedback level and the motor control tests (Table 4).

The estimates of the ICC_1,2_ for the inter-tester reliability of the motor control and biofeedback level assessment procedure across the two therapists were satisfactory (Table 5), ranging from 0.55 to 0.86 except for hook lying and sitting knee extension, which scored lower (0.39 and 0.31, respectively).

## 4. Discussion

The present study applied motor learning principles to TrA activation/contraction capacity during an 8-week progressive motor control exercise program, investigating whether visual US-guided feedback is more effective in rehabilitation than the traditional rehabilitation methods with palpatory cues. The results from this pilot study showed that there were no statistically significant differences in all outcome measures between the intervention and control groups; thus, not proving that US-guided biofeedback is superior to traditional palpatory cuing. However, as far as the entire sample is concerned, it appears that there were statistically significant improvements in all outcomes following the intervention, except for the HADS anxiety scale, in which there were no differences. 

Though our sample was small, and no conclusion can be made with confidence, it is comparable with former studies as far as gender (reporting that women are more likely to experience back pain), disability, functionality and study outcome are concerned [8,14,18,25,49]. 

Six progressive levels of TrA activation were developed for the study, demonstrating good inter-examiner reliability. Also, as no ceiling effects were scored by our sample, the scale’s feasibility and usefulness are supported. Most participants before the intervention were at an average level of TrA activation (52.2% were at Level 3 and 39.1% were at Level 4). Despite previous studies [3] reporting difficulties in activating TrA prior to training, in our sample this was not the case. 

The addition of real-time US as a feedback-guided aid to the motor learning process has been used in previous studies [10,18,36,50,51,52,53,54,55]. In some of them, it has been shown to be superior to other methods (such as tactile or verbal feedback) [5]. However, this was not confirmed in our study. After the intervention program, there was a significant improvement in the level of TrA activation across both groups, indicating that the type of feedback provided during the exercise program was equally effective across the groups [18,36]; the intervention group, which received visual feedback using the US did not appear to outperform. Our results may be partly explained by the fact that the initial TrA activation level was satisfactory at baseline across our sample; thus, feedback may have not been as necessary. Additionally, the fact that the control group did receive tactile feedback, according to traditional (classic) physiotherapy session delivery methods, may also explain the results.

These results partly agree with the study by Van et al. [50], in which 25 healthy subjects, randomly divided into two groups, received different feedback (one group received visual feedback by watching the multifidus muscle contract using the US plus verbal feedback, and the other group received only verbal feedback); subjects from both groups equally increased their multifidus muscle activation (however, the US feedback group retained more of the motor skill than those receiving verbal feedback alone). Another study by Henry and Westervelt [18], in which three groups of healthy subjects were evaluated for TrA activation (verbal feedback group, verbal and tactile feedback group, US-guided visual feedback group), showed that the US group had better results for TrA activation, requiring significantly fewer trials to reach the performance criterion, however, no differences among groups were found long-term. Both studies, however, used healthy asymptomatic samples. From the above, we conclude that further research on patient samples with no TrA activation capacity would be of great interest in investigating US-guided feedback. Nevertheless, the addition of rehabilitative ultrasound imaging (RUSI) to the implementation of the motor control exercise program facilitates both the therapist, who has a picture of the deeper muscle contractions in real time and the patient, who seems to understand better and much faster what to do. 

Regarding the whole sample, showing improvements in all outcomes measured, our results confirm two previous studies [18,52] on the therapeutic benefits of providing feedback in motor learning. Also, the results of this study come to confirm the fact that the application of motor control exercises (MCE) can bring about an improvement both in the levels of pain and in the levels of functionality for the individual. These results are in agreement with previous studies [16,56,57,58], demonstrating positive results both for improving pain and function in their samples. According to Ferreira et al. [59], the effects of treatment with motor control exercises are greater in subjects suffering from NSLBP and showing poorer TrA activation. It has been debated whether MCE should focus on the isolated contraction of the local musculature or whether exercises should aim at activating all abdominal and back extensor muscles to ensure spinal stability and endurance [60], as recent research shows that there is increased activation of the deep abdominals in functional and weight-bearing postures [13,61,62].

It is not yet known whether the improvements of MCE on pain and physical disability in NSLBP are due to the isolated activation of the local musculature or to the later stages of the intervention, where, loaded postures to co-contracting trunk muscles are being involved. Isolated contraction of the local muscles, however, appears necessary to restore disrupted local musculature activation patterns in low back pain populations [63,64]. From the above, it is concluded that further research is needed to investigate the underlying mechanisms of the effect of these exercises on pain and functional limitations.

Clinical Relevance: The results of the present research revealed that both groups improved their level of achievement regardless of the kind of feedback that was provided; any kind of feedback given (either tactile or via RUSI) is an effective tool for physiotherapists to use clinically for the activation of the TrA during motor skill training [53]. Principles of motor learning can be used to explain why visual feedback is beneficial for people with NSLBP. Clinicians have highlighted the difficulty that patients face when trying to selectively activate deep abdominal musculature (e.g., TrA) [65]. This may be due to processes such as reflex inhibition [66]; and, since individuals with low back pain have been shown to have reduced proprioception, which affects their ability to provide and process endogenous feedback, increased extrinsic visual feedback may be indicated [67]. Our conclusions are consistent with motor learning principles, however, they do not acknowledge the superiority of visual feedback over classic tactile feedback. 

Limitations and future suggestions: The study, in view of being a preliminary (pilot) one, included a relatively small sample, and, even if it resembled a typical chronic NSLBP patient sample, the validity of our findings is limited. Also, both groups had a fair TrA contraction at baseline; thus, possibly limiting the effectiveness of the intervention. Another shortcoming is that the results were assessed immediately after the intervention, without providing follow-up, though previous studies have shown maintenance of improvement in subsequent long-term reassessments [51,53,54].

Future studies should also target patient samples with no or minimal TrA activation. Also, larger samples and the training of TrA activation using real-time ultrasound during functional skills (e.g., work, sports) are needed. Electromyographic measurement to record changes during training and during test performance would add to the validity of our findings and its use in muscle activation; only one study has used electromyography as a means of post-training assessment combined with RUSI feedback [55]. Finally, we also believe that TrA activation training programs that apply motor learning principles (e.g., feedback using RUSI) [68] and offer activity-specific training will improve learning and may produce greater compliance with respect to the work environment [54].

## 5. Conclusions

Both, the US-guided feedback group and the control (traditional intervention) yielded statistically significant improvements (*p* > 0.05) post-intervention in all measured variables (pain, pressure stabilizer scores, motor control disability, anxiety and depression). However, there were no significant group x time interactions for any of the outcomes, thus, not indicating any superiority of the US-guided group against the control.

These findings, though cannot be generalized, confirm the benefits of motor control exercises in reducing pain, improving function, psychosocial levels, motor control and TrA muscle activation across our NSLBP sample. The results of this pilot confirm the connection of deficient motor control with NSLBP and that the treatment of these disturbances focusing on motor control exercise programs is important. Future studies should focus on exploring optimal feedback type on patient samples with minimal or no TrA or other deep spinal muscle activation.

## Figures and Tables

**Table 1:** Baseline demographic and clinical characteristics for each group.

	US Group (*n* = 12)		Control Group (*n* = 11)		Between Group Differences
	Mean (SD)	95% CI (Lower–Upper Bound)	Mean (SD)	95% CI (Lower-Upper Bound)	*p*-Value
Age (years)	47.67 (8.86)	42.04–53.29	47.18 (14.56)	37.40–56.96	0.923
Height (cm)	168.83 (9.49)	162.81–174.86	166.73 (8.95)	160.72–172.74	0.590
Weight (kg)	4.08 (3.15)	2.08–6.08	3.73 (3.29)	1.52–5.94	0.793
BMI (kg/m^2^)	2.75 (0.45)	2.46–3.04	2.82 (0.75)	2.31–3.32	0.915
Gender	Frequency (Percentage)				
Male	4 (33.3%)		2 (18.2%)		
Female	8 (66.7%)		9 (81.8%)		0.408

SD = standard deviation, BMI = body mass index, *n* = number of participants, CI = Confidence Interval.

**Table 2:** Baseline and post-intervention results for the whole sample (*n* = 23).

	Baseline	Post Intervention	Independent *t*
	Mean Value (SD)		*p*-Value
NPRS (at worst)	7.87 (1.74)	2.61 (1.75)	<0.001 **
NPRS (at best)	1.87 (1.60)	0.13 (0.46)	<0.001 **
NPRS (leg pain)	4.04 (4.09)	1.00 (1.54)	<0.001 **
RMDQ	9.91 (5.06)	2.43 (2.43)	<0.001 **
HADS-Anxiety	7.52 (3.85)	7.04 (3.90)	0.460
HADS-Depression	5.39 (3.04)	3.91 (3.15)	0.023 *
	Frequency (Percentage)		*X* ^2^
TrA biofeedback level			
Level 1	0 (0%)	0 (0%)	0.017 *
Level 2	1 (4.3%)	0 (0%)	
Level 3	12 (52.2%)	7 (30.4%)	
Level 4	9 (39.1%)	8 (34.8%)	
Level 5	0 (0%)	5 (21.7%)	
Level 6	1 (4.3%)	3 (13%)	
Motor Control tests			
Waiter’s bow	12 (52.2%)	21 (91.3%)	0.004 *
Pelvic tilt	15 (65.2%)	22 (95.7%)	0.010 *
Hook lying	3 (13%)	21 (91.3%)	<0.001 **
Quadruped (flexion-control)	9 (39.1%)	19 (82.6%)	0.003 *
Quadruped (extension-control)	5 (21.7%)	17 (73.9%)	<0.001 **
Active knee flexion in (prone)	15 (65.2%)	23 (100%)	0.002 *
Sitting knee extension	18 (78.3%)	23 (100%)	0.019 *

NPRS = Numerical Pain Rate Scale, RMDQ = Roland Morris Disability Questionnaire, HADS = Hospital Anxiety and Depression Scale. * *p* is statistically significant (<0.05). ** *p* is highly significant (<0.001).

**Table 3:** Baseline and post-intervention outcomes on pain, disability, anxiety and depression across groups.

	US-Guided Group (*n* = 12)		Control Group (*n* = 11)		
	Baseline	Final	Baseline	Final	Two-Way Anova
	Mean Value (SD)				*p*-Value
NPRS (worst)	7.33 (1.97)	2.83 (1.70)	8.45 (1.29)	2.36 (1.86)	0.593
NPRS (best)	1.75 (1.71)	0.08 (0.30)	2 (1.55)	0.18 (0.60)	0.655
NPRS (leg pain)	4.42 (4.70)	1.33 (1.83)	3.64 (3.50)	0.64 (1.12)	0.425
RMDQ	8.75 (4.52)	2.83 (2.33)	11.18 (5.53)	2 (2.57)	0.529
HADS-Anxiety	6.58 (4.21)	6.75 (3.96)	8.55 (3.30)	7.36 (4.01)	0.329
HADS-Depression	5.92 (2.97)	4.08 (3.29)	4.82 (3.16)	3.73 (3.29)	0.539

NPRS = Numerical Pain Rate Scale, RMDQ = Roland Morris Disability Questionnaire, HADS = Hospital Anxiety and Depression Scale, MCT = motor control test.

**Table 4:** Results on TrA biofeedback assessment level and motor control tests across groups.

	Baseline		Post-Intervention		
Clinical Tests	US-Guided	Control Group	US-Guided	Control Group	
TrA Biofeedback Level	Frequency (Percentage)		Frequency (Percentage)		*p*-Value
Level 1	0 (0%)	0 (0%)	0 (0%)	0 (0%)	0.898
Level 2	0 (0%)	1 (9.1%)	0 (0%)	0 (0%)	
Level 3	4 (33.3%)	8 (72.7%)	4 (33.3%)	3 (27.3%)	
Level 4	8 (66.7%)	1 (9.1%)	4 (33.3%)	4 (36.4%)	
Level 5	0 (0%)	0 (0%)	2 (16.7%)	3 (27.3%)	
Level 6	0 (0%)	1 (9.1%)	2 (16.7%)	1 (9.1%)	
Motor Control Tests					
Waiters bow	6 (50.0%)	6 (54.5%)	11 (91.7%)	10 (90.9%)	0.950
Pelvic tilt	8 (66.7%)	7 (63.6%)	12 (100%)	10 (90.9%)	0.296
Hook lying	3 (25.0%)	0 (0%)	12 (100%)	9 (81.8%)	0.131
Quadruped (flexion-control)	5 (41.7%)	4 (36.4%)	11 (91.7%)	9 (75.0%)	0.242
Quadruped (extension-control)	4 (33.3%)	1 (9.1%)	9 (75.0%)	9 (75.0%)	0.903
Active knee flexion (prone)	9 (75.0%)	6 (54.5%)	12 (100%)	11 (100%)	1.000
Sitting knee extension	9 (75.0%)	9 (81.8%)	12 (100%)	11 (100%)	1.000

**Table 5:** Reliability results based on Intraclass Correlation Coefficients (ICC_1,2_) for the Biofeedback level assessment procedure and the Motor control tests.

	ICC_1,2_ Value
Biofeedback level assessment procedure	0.86
Motor Control Tests	
Waiters bow	0.75
Pelvic tilt	0.65
Hook lying position	0.39
Quadruped (flexion control)	0.55
Quadruped (extension control)	0.72
Active knee flexion (in prone)	0.66
Sitting knee extension	0.31

## Data Availability

The data presented in this study are available on request from the corresponding author. The data are not publicly available due to privacy restrictions.

## Appendix A

**Warm-up Phase** *3 sets of 10 repetitions*		
Intervention/Details	Figures	
(1) Patient lies on his back with bent legs and tries to perform a posterior pelvic tilt.		
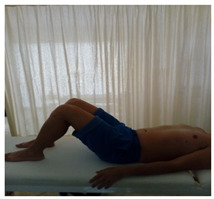
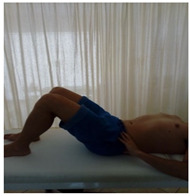
(2) Patient lies on his back with bent legs, and takes each leg with both hands and brings it towards the chest alternately.	**	**
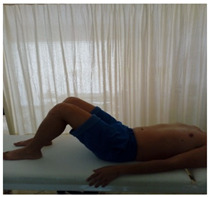
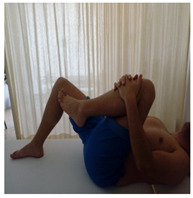
(3) Patient lies on his back with bent legs bent and lets them fall left and right together.		
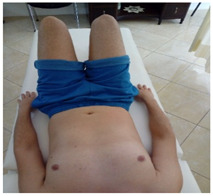
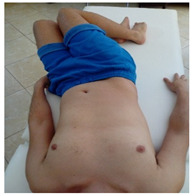
(4) From a supine position with the legs loosely stretched the patient tries to lengthen each leg from the pelvis		
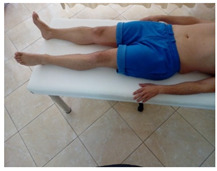
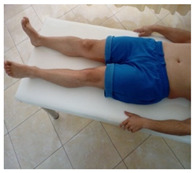
(5) From a prone position with hands clasped behind the waist, the person lifts the upper body and head slightly from the bed.		
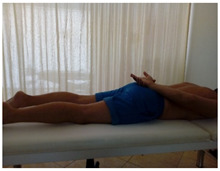
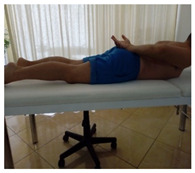
(6) From a quadruped position, hands on shoulder height and knees onhip height, patient perform the “cat-camel” exercise.		
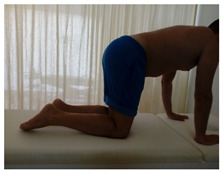
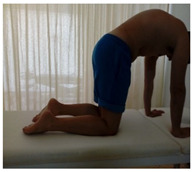

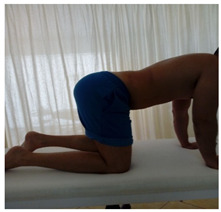
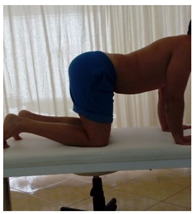
	
**Main Motor Control Exercise Program** *3 sets of 10 repetitions*		
Intervention/Details	Figures	
(1) From a supine position with bent legs the patient tries to bring the navel towards the spine by tightening lower abdomen (static control).		
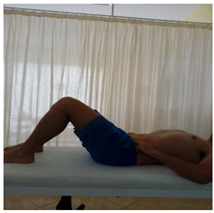
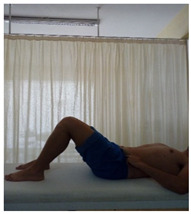
(2) TrA contraction (from crook linyg) while leting the leg to fallout to the side and back (initial position), where contraction relaxes. Leg bends out as far as patient feels can control TrA contraction, leg and pelvis (roation control).		
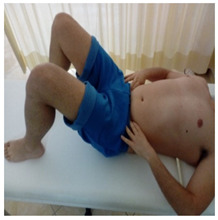
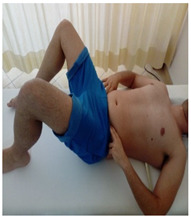
(3) TrA contraction (from crook linyg) while lifting the leg from the bed and returning it to the initial position (where contraction is relaxed). Leg bends slowly as far as patient feels can control TrA contraction, leg and pelvis (flexion control).		
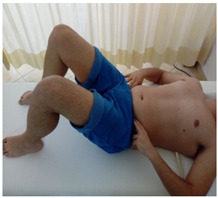
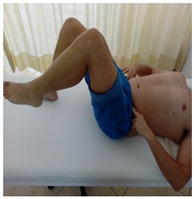
(4) TrA contraction (from crook linyg) while dragging the leg from the bed without losing contact with the ground and back (full knee extension is avoided). Leg is stretched out as far as patient feels can control TrA contraction, leg and pelvis (extension control).		
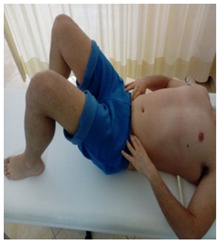
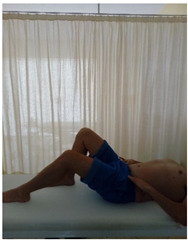
(5) TrA contraction in prone with alternative knee flexion to 90°. Contraction relaxes when leg returns to the starting position (extension/rotation control).		
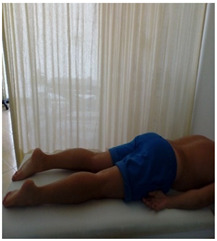
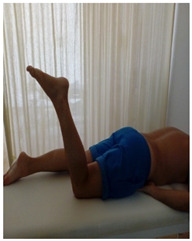
(6) TrA contraction from quadrupedal with hands below shoulder height and knees below hips. Patient brings trunk forward passing over his hands while trying to keep the waist flat. The contraction is relaxed on return to starting position (flexion control).		
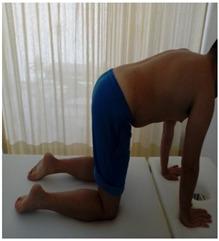
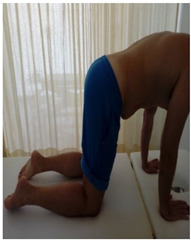
(7) TrA contraction from quadrupedal (as above). Patient brings the trunk towards his legs while trying to keep the waist flat. The contraction is relaxed on return to starting position (extension control).		
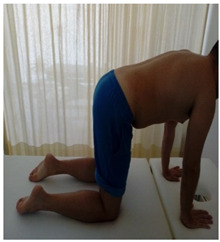
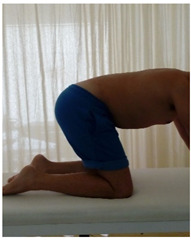
(8) TrA contraction in sitting with the body upright and the arms relaxed by the side of the body. Patient extends knee slowly (but not fully) whistle trying th maintain TrA contraction and posture. Contraction is relaxed by returning the leg to the starting position (partial weight-bearing flexion control).		
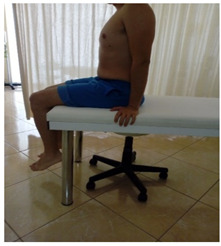
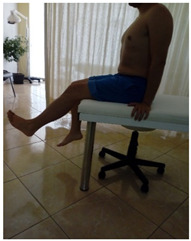
(9) TrA contraction in sitting with the body upright and the arms relaxed by the side of the body. Patient flexes the hip whistle trying th maintain TrA contraction and posture. The contraction is relaxed by returning the leg to the starting position (partial weight-bearing flexion control).		
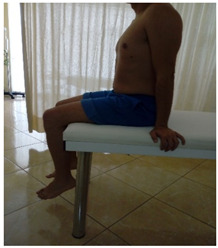
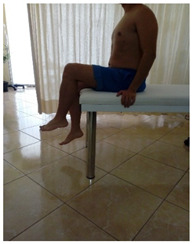
(10) Standing resting comfortable the spine on the wall with knees flexed away from the wall. TrA contraction while the patient performed a posterio pelvic tilt. The contraction is relaxed by returning to the starting position (weight-bearing flexion control).		
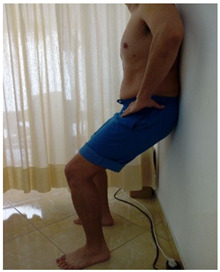
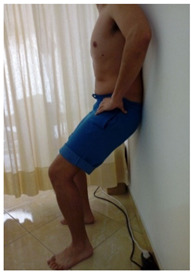
(11) TrA contraction in standing. Patient tries to bend forwards (bowing) trying to keep the waist straight (flat). Trunk flexion should occur from the hips (not from the waist). Hands can be used for additional support. The contraction is relaxed by returning to the starting position (weight-bearing flexion/extension control).		
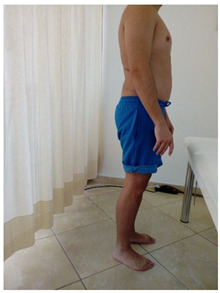
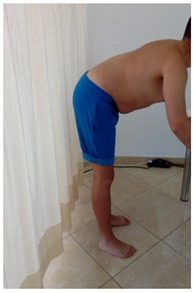
**Stretching Exercises (recovery phase)** *60 s/3 repetitions*		
Intervention/Details	Figures	
(1) Supine position with the legs relaxed, the patient grabs one leg by the knee and brings it to the chest and holds (low lumber muscle stretch).		
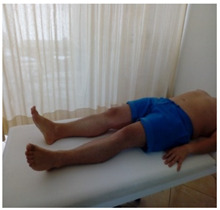
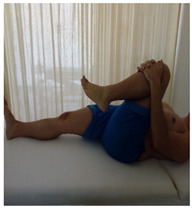
(2) Supine position with the legs relaxed, the patient bends one leg to touch the opposite side of the bed and lets it drop. At the same time he turns his head to the opposite side (low lumber side muscle stretch).		
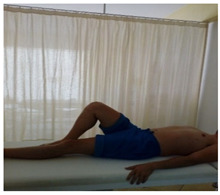
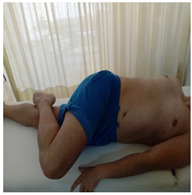
(3) Supine position with the legs bent, the patient crosses one leg over the other and with both hands grasps the leg resting on the bed and brings it towards the chest (piroformis stretch).		
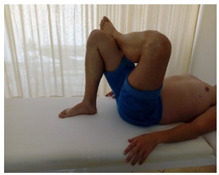
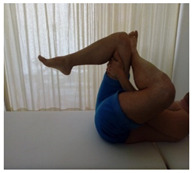
(4) Supine position with the legs relaxed and using a belt the patient brings the leg outstretched into flexion until a pull is felt on the back of the leg and holds it firmly there (hamstring stretch).		

## References

[1] Jeffries L.J., Milanese S.F., Grimmer K.A. (2007). Epidemiology of Adolescent Spinal Pain: A systematic overview of the research literature. Spine.

[2] Balagué F., Mannion A.F., Pellisé F., Cedraschi C. (2012). Non-specific low back pain. Lancet.

[3] Hodges P.W., Richardson C., Jull G. (1996). Evaluation of the relationship between laboratory and clinical tests of transverses abdominus function. Physiother. Res. Int..

[4] Bergmark A. (1989). Stability of the lumbar spine: A study in mechanical engineering. Acta Orthop. Scand. Suppl..

[5] Richardson C.A., Hodges P.W., Hides J.A. (2004). Therapeutic Exercise for Spinal Segmental Stabilization: A Motor Control Approach for the Treatment and Prevention of Low Back Pain.

[6] Hodges P.W., Richardson C.A. (1998). Delayed postural contraction of transversus abdominis in low back pain associated with movement of the lower limb. J. Spinal Disord. Tech..

[7] Panjabi M.M. (1992). The stabilizing system of the spine. Part II. Neutral zone and instability hypothesis. J. Spinal Disord. Tech..

[8] Akbari A., Khorashadizadeh S., Abdi G. (2008). The effect of motor control exercise versus general exercise on lumbar local stabilizing muscles thickness: Randomized controlled trial of patients with chronic low back pain. J. Back Musc. Rehab..

[9] Mannion A.F., Pulkovski N., Toma V., Sprott H. (2008). Abdominal muscle size and symmetry at rest and during abdominal hollowing exercises in healthy control subjects. J. Anat..

[10] Hides J.A., Richardson C.A., Jull G.A. (1996). Multifidus muscle recovery is not automatic after resolution of acute, first-episode low back pain. Spine.

[11] Hodges P.W., Richardson C.A. (1999). Altered trunk muscle recruitment in people with low back pain with upper limb movement at different speeds. Arch. Phys. Med. Rehabil..

[12] Hides J.A., Jull G.A., Richardson C.A. (2001). Long-term effects of specific stabilizing exercises for first-episode low back pain. Spine.

[13] Crommert M.E., Ekblom M.M., Thorstensson A. (2011). Activation of transversus abdominis varies with postural demand in standing. Gait Posture.

[14] Lamoth C.J.C., Meijer O.G., Daffertshofer A., Wuisman P.I.J.M., Beek P.J. (2006). Effects of chronic low back pain on trunk coordination and back muscle activity during walking: Changes in motor control. Eur.Spine J..

[15] Bystrom M.G., Rasmussen-Barr E., Grooten W.J.A. (2013). Motor control exercises reduces pain and disability in chronic and recurrent low back pain. Spine.

[16] O’Sullivan P.B., Phyty G.D., Twomey L.T., Allison G.T. (1997). Evaluation of specific stabilizing exercise in the treatment of chronic low back pain with radiologic diagnosis of spondylolysis or spondylolisthesis. Spine.

[17] Hides J., Richardson C.A., Jull G.A., Davies S. (1995). Ultrasound imaging in rehabilitation. Aust. J. Physiother..

[18] Henry S.M., Westervelt K.C. (2005). The use of real-time ultrasound feedback in teaching abdominal hollowing exercises to healthy subjects. J. Orthop. Sports Phys. Ther..

[19] Weiser S., Rossignol M. (2006). Triage for Nonspecific Lower-back Pain. Clin. Orthop. Relat. Res..

[20] Lizier D.T., Perez M.V., Sakata R.K. (2012). Exercises for Treatment of Nonspecific Low Back Pain. Rev. Bras. Anestesiol..

[21] Joaquim A. (2016). Initial approach to patients with acute lower back pain. Rev. Assoc. Méd. Bras..

[22] Falla D., Bilenkij G., Jull G. (2004). Patients with chronic neck pain demonstrate altered patterns of muscle activation during performance of a functional upper limb task. Spine.

[23] Hodges P.W., Moseley L.G. (2003). Pain and motor control of the lumbopelvic region: Effect and possible mechanisms. J. Electromyogr. Kinesiol..

[24] Shumway-Cook A., Woollacott M.H. (2016). Motor Control: Translating Research into Clinical Practice.

[25] Ferreira M.L., Ferreira P.H., Latimer J., Herbert R.D., Hodges P.W., Jennings M.D., Maher C.G., Refshauge K.M. (2007). Comparison of general exercise, motor control exercise and spinal manipulative therapy for chronic low back pain: A randomized trial. Pain.

[26] Costa L.O., Maher C.G., Latimer J., Hodges P.W., Herbert R.D., Refshauge K.M., McAuley J.H., Jennings M.D. (2009). Motor control exercise for chronic low back pain: A randomized placebo-controlled trial. Phys. Ther..

[27] Franca F.R., Burke T.N., Hanada E.S., Marques A.P. (2010). Segmental stabilization and muscular strengthening in chronic low back pain—A comparative study. Clinics.

[28] Fradkin A.J., Zazryn T.R., Smoliga J.M. (2010). Effects of warming-up on physical performance: A systematic review with meta-analysis. J. Strength Cond. Res..

[29] Sahrmann S.A. (2002). Diagnosis and Treatment of Movement Impairment Syndromes.

[30] O’Sullivan P.B. (2005). Diagnosis and classification of chronic low back pain disorders: Maladaptive movement and motor control impairments as underlying mechanism. Man. Ther..

[31] Okragly R., Micheli L.J. (2011). Static Stretching. Encyclopedia of Sports Medicine.

[32] McHugh M., Cosgrave C. (2010). To stretch or not to stretch: The role of stretching in injury prevention and performance. Scand. J. Med. Sci. Sports.

[33] Costa L.O.P., da Cunha Menezes Costa L., Cançado R.L., De Melo Oliveira W., Ferreira P.H. (2006). Intra-tester reliability of two clinical tests of transversus abdominis muscle recruitment. Physiother. Res. Int..

[34] Whittaker J.L. (2007). Ultrasound Imaging for Rehabilitation of the Lumbopelvic Region: A Clinical Approach.

[35] McMeeken J.M., Beith I.D., Newham D.J., Milligan P., Critchley D.J. (2004). The relationship between EMG and change in thickness of transversus abdominis. Clin. Biomech..

[36] Teyhen D.S., Miltenberger C.E., Deiters H.M., del Toro Y.M., Pulliam J.N., Childs J.D., Boyles R.E., Flynn T.W. (2005). The use of ultrasound imaging of the abdominal drawing-in maneuver in subjects with low back pain. J. Orthop. Sports Phys. Ther..

[37] Childs J.D., Piva S.R., Fritz J.M. (2005). Responsiveness of the numeric pain rating scale in patients with low back pain. Spine.

[38] Roland M., Morris R. (1983). A study of the natural history of back pain. Part I: Development of a reliable and sensitive measure of disability in low-back pain. Spine.

[39] Zigmond A.S., Snaith R.P. (1983). The hospital anxiety and depression scale. Acta Psychiatr. Scand..

[40] Luomajoki H., Kool J., de Bruin E.D., Airaksinen O. (2007). Reliability of movement control tests in the lumbar spine. BMC Musculosk. Disord..

[41] Cole B., Finch E., Gowland C., Mayo N., Basmajian J. (1994). Physical Rehabilitation Outcome Measures.

[42] Richardson C., Jull G., Hodges P., Hides J. (1999). Therapeutic Exercise for Spinal Segmental Stabilisation in Low Back Pain, Scientific Basis and Clinical Approach.

[43] Cairns M.C., Foster N.E., Wright C. (2006). Randomized controlled trial of specific spinal stabilization exercises and conventional physiotherapy for recurrent low back pain. Spine.

[44] Costa L.O.P., Costa L.C.M., Cançado R.L., Oliveira W.M., Ferreira P.H. (2004). Confiabilidade do teste palpatório e da unidade de biofeedback pressórico na ativação do músculo transverso abdominal em indivíduos normais. Acta Fisiátrica.

[45] Chattanooga G. (2005). Stabilizer Pressure Bio-Feedback. Operating Instructions.

[46] Lima P., Oliveira R., Moura Filho A., Raposo M., Costa L., Laurentino G. (2012). Concurrent validity of the pressure biofeedback unit and surface electromyography in measuring transversus abdominis muscle activity in patients with chronic nonspecific low back pain. Rev. Bras. Fisioter..

[47] Chon S.C., Chang K.Y., You J.S. (2010). Effect of the abdominal drawin manoeuvre in combination with ankle dorsiflexion in strengthening the transverse abdominal muscle in healthy young adults: A preliminary, randomised, controlled study. Physiotherapy.

[48] Lee S.H., Kim T.H., Lee B.H. (2014). The effect of abdominal bracing in combination with low extremity movement son changes in thickness of abdominal muscles and lumbar strength for low back pain. J. Phys. Ther. Sci..

[49] Schneider S., Randoll D., Buchner M. (2006). Why do women have back pain more than men? A representative prevalence study in the federal republic of Germany. Clin. J. Pain.

[50] Van K., Hides J.A., Richardson C.A. (2006). The use of real-time ultrasound imaging for biofeedback of lumbar multifidus muscle contraction in healthy subjects. J. Orthop. Sports Phys. Ther..

[51] Kermode F. (2004). Benefits of utilizing real-time ultrasound imaging in the rehabilitation of the lumbar spine stabilizing muscles following low back injury in the elite athlete—A single case study. Phys. Ther. Sport.

[52] Worth S., Henry S.M., Bunn Y. (2007). Real-time ultrasound feedback and abdominal hollowing exercises for people with back pain. N. Z. J. Physiother..

[53] Herbert W.J., Heiss D.G., Basso D.M. (2008). Influence of feedback schedule in motor performance and learning of a lumbar multifidus muscle task using rehabilitative ultrasound imaging: A randomized clinical trial. Phys. Ther..

[54] McPherson S.L., Watson T. (2014). Training of transversus abdominis activation in the supine position with ultrasound biofeedback translated to increased transversus abdominis activation during upright loaded functional tasks. PM&R.

[55] Chen Y.H., Chai H.M., Yang J.L., Lin Y.J., Wang S.F. (2015). Reliability and validity of Transversus Abdominis measurement at the posterior muscle-fascia junction with ultrasonography in asymptomatic participants. J. Manip. Physiol. Ther..

[56] Moseley G.L., Hodges P.W., Gandevia S.C. (2002). Deep and superficial fibers of the lumbar multifidus muscle are differentially active during voluntary arm movements. Spine.

[57] Goldby L.J., Moore A.P., Doust J., Trew M.E. (2006). A randomized controlled trial investigating the efficiency of musculoskeletal physiotherapy on chronic low back disorder. Spine.

[58] Macedo L.G., Maher C.G., Latimer J., McAuley J.H. (2009). Motor control exercise for persistent, nonspecific low back pain: A systematic review. Phys. Ther..

[59] Ferreira P.H., Ferreira M.L., Maher C.G., Refshauge K., Herbert R.D., Hodges P.W. (2010). Changes in recruitment of transversus abdominis correlate with disability in people with chronic low back pain. Br. J. Sports Med..

[60] McGill S. (2007). Low Back Disorders: Evidence-Based Prevention and Rehabilitation.

[61] Rasouli O., Arab A.M., Jaberzadeh S. (2011). Ultrasound measurement of deep abdominal muscle activity in sitting positions with different stability levels in subjects with and without chronic low back pain. Man. Ther..

[62] Pinto R.F., Ferreira P.H., Franco M.R., Ferreira M.C., Ferreira M.L., Teixeira-Salmela L.F., Oliveira V.C., Maher C. (2011). The effect of lumbar posture on abdominal muscle thickness during an isometric leg task in people with and without non-specific low back pain. Man. Ther..

[63] Tsao H., Hodges P.W. (2007). Immediate changes in feed forward postural adjustments following voluntary motor training. Exp. Brain Res..

[64] Hodges P.W. (2011). Pain and motor control: From the laboratory to rehabilitation. J. Electromyogr. Kinesiol..

[65] Hides J.A., Richardson C.A., Jull G.A. (1998). Use of real-time ultrasound imaging for feedback in rehabilitation. Man. Ther..

[66] Hides J.A., Stokes M.J., Saide M., Jull G.A., Cooper D.H. (1994). Evidence of lumbar multifidus muscles wasting ipsilateral to symptoms in patients with acute/subacute low back pain. Spine.

[67] Parkhurst T.M., Burnett C.N. (1994). Injury and proprioception in the lower back. J. Orthop. Sports Phys. Ther..

[68] Jarus T., Ratzon N.Z. (2005). The implementation of motor learning principles in designing prevention programs at work. Work.

